# Probabilistic modeling and machine learning in structural and systems biology

**DOI:** 10.1186/1471-2105-8-S2-S1

**Published:** 2007-05-03

**Authors:** Samuel Kaski, Juho Rousu, Esko Ukkonen

**Affiliations:** 1 Adaptive Informatics Research Centre and Helsinki Institute for Information Technology, Laboratory of Computer and Information Science, Helsinki University of Technology, P.O. Box 5400, FI-02015 TKK, Finland; 2 Helsinki Institute for Information Technology, Department of Computer Science, University of Helsinki, P.O. Box 68, FI-00014 University of Helsinki, Finland

## Abstract

This supplement contains extended versions of a selected subset of papers presented at the workshop PMSB 2007, Probabilistic Modeling and Machine Learning in Structural and Systems Biology, Tuusula, Finland, from June 17 to 18, 2006.

## Introduction

The workshop was designed to gather together researchers working on the extremely timely task of integrating advanced machine learning and computational modeling with current biological and medical research problems. The field is particularly active because the new high-throughput measurement techniques and biological databases require advanced modeling methods but the field progresses so rapidly that the models need to be flexible and relatively general-purpose. Modeling approaches on both the systems level and in structural biology have already become a necessary part of normal research practice. While the combination of machine learning and biological research is a particularly good match with lots of opportunities, the work requires expertise in several areas and hence is very challenging and needs frequent interaction between researchers.

The group of researchers working in this field is normally spread thinly in the currently abundant, partly but not fully relevant conferences: both biological and bioinformatics conferences on the one hand, and pure machine learning conferences on the other. The aim of this workshop was to function as a specifically targeted forum.

The workshop started a series which will be continued as MLSB'07, Machine Learning for Structural and Systems Biology, June 28–29, in Evry, France.

## Summary of the supplement

Selected submissions were invited based on the papers presented in the workshop. We targeted a subset of around ten best papers, and almost succeeded. This supplement contains a reviewed selection of eleven full papers.

Two of the papers are about modeling of the causal or physical behavior of cellular systems. Rogers et al. [1] introduce a full-Bayesian model of kinetics of the activity of transcription factors in gene regulation, and Opgen-Rhein and Strimmer [2] use shrinkage methods to estimate autoregressive processes from small samples, to infer causal gene regulatory networks.

Biological networks are modeled in three further papers as well. Geurts et al. [3] predict links in protein-protein interaction networks and enzyme networks with a new kind of kernel-based method. Michoel et al. [4] use a synthetic data generator to evaluate the performance of methods for learning module networks, including a new one they introduce.

Analysis of high-throughput data is a common subtheme in most works. Three of the papers are particularly focused in this task. Yoon et al. [5] introduce a robust preprocessing method for treating missing values in gene expression data, and Bertoni and Valentini [6] decide the number of clusters based on stability against fluctuations caused by random projections. In the only paper on metabonomics, Vehtari et al. [7] introduce a full-Bayesian way of modeling the mapping between NMR spectra and clinical variables. Three of the papers are related to genomics. Landwehr et al. [8] introduce a hidden Markov model-based method for haplotype reconstruction which is a subproblem of gene association studies for uncovering genetic bases of diseases. Dix et al. [9] use compression methods to analyze information content of DNA in a genome-wide scale. Oja et al. [10] use hidden Markov model-based methods to estimate activities of retroviruses residing in human genome, using EST databases.

Finally, Roth and Fischer [11] introduce a kernel-based fusion from multiple data sources for predicting multi-label protein function.
